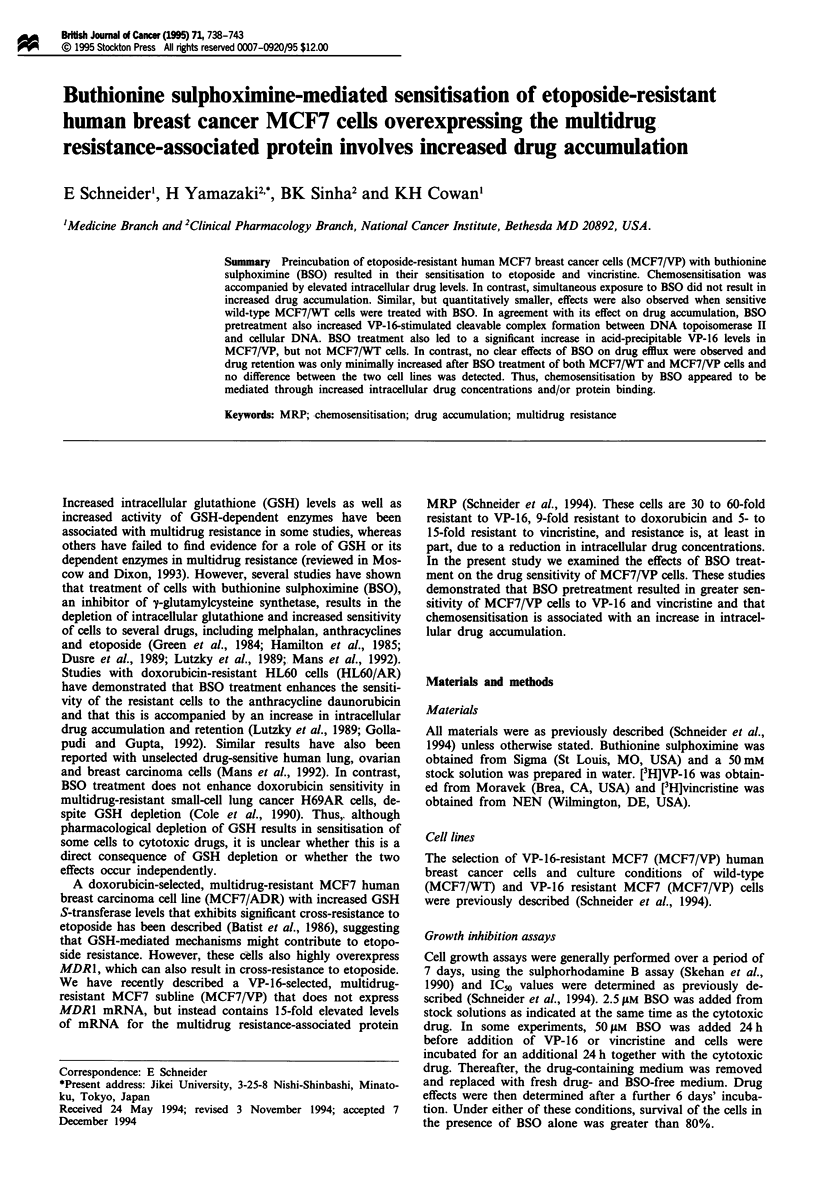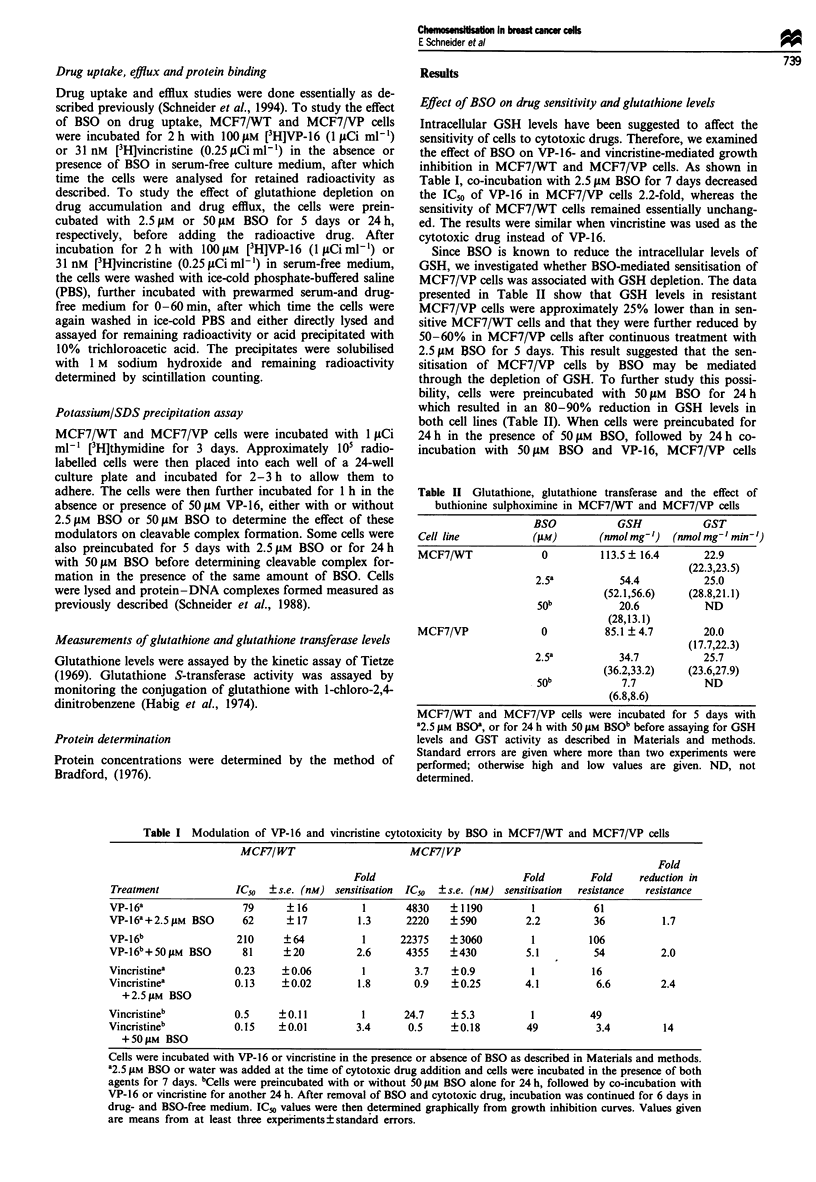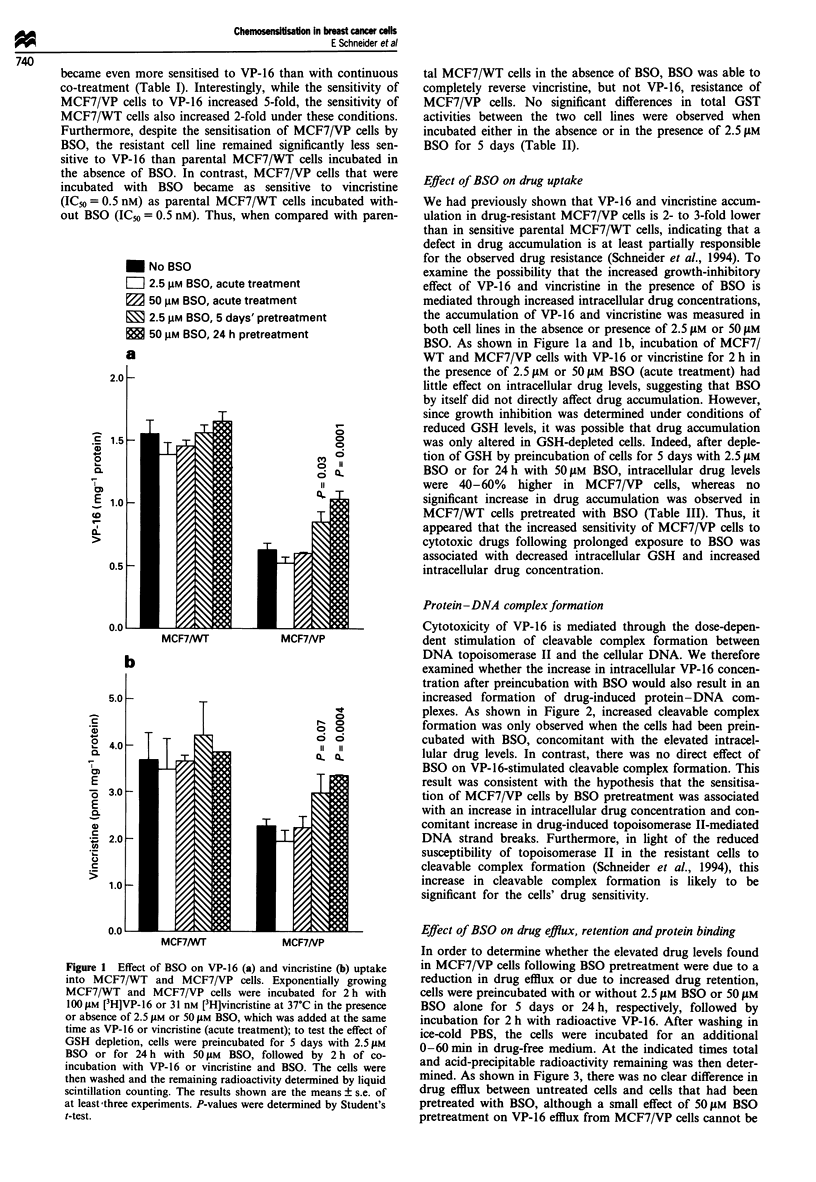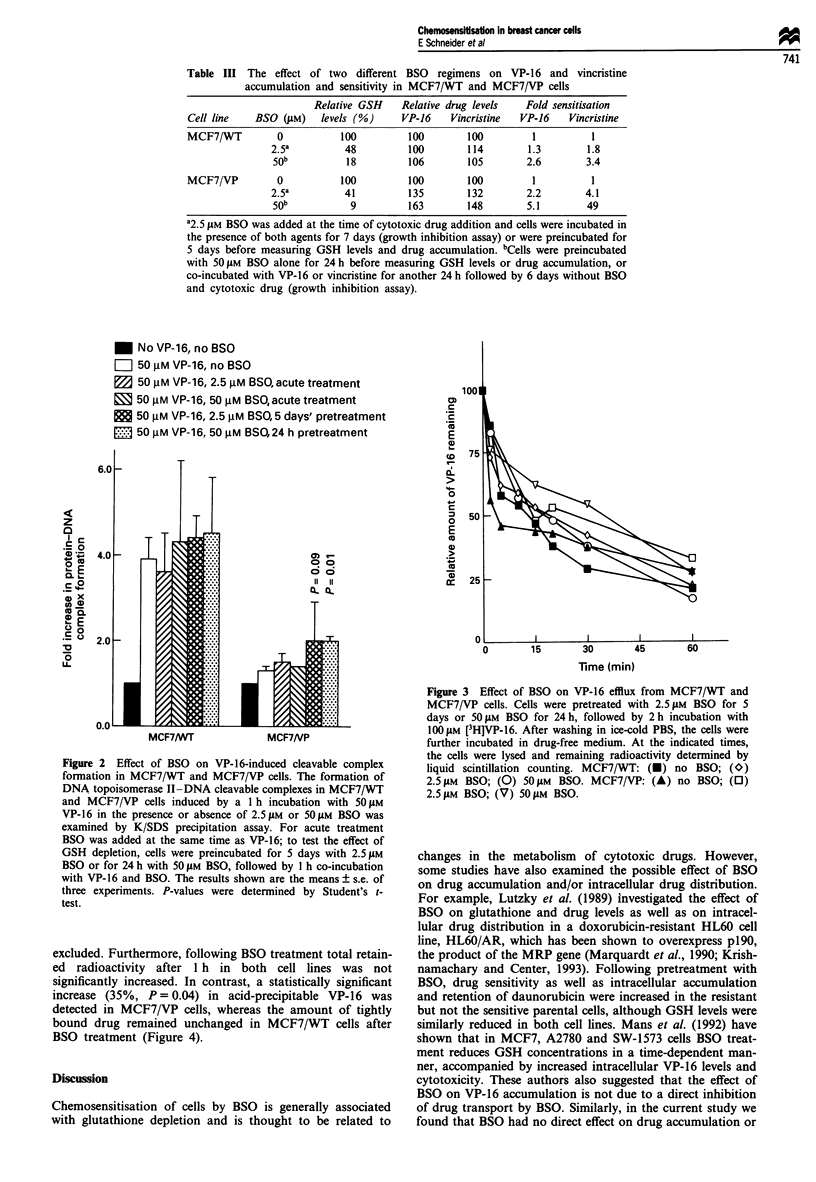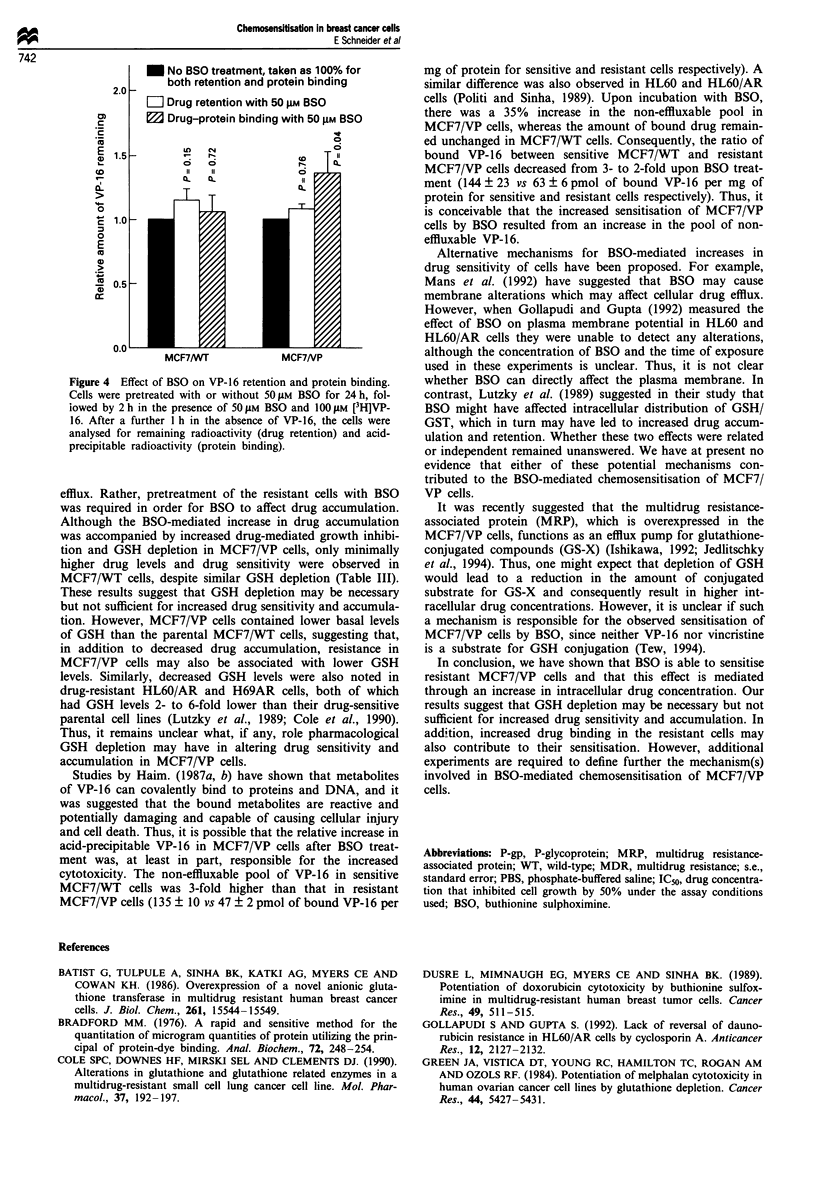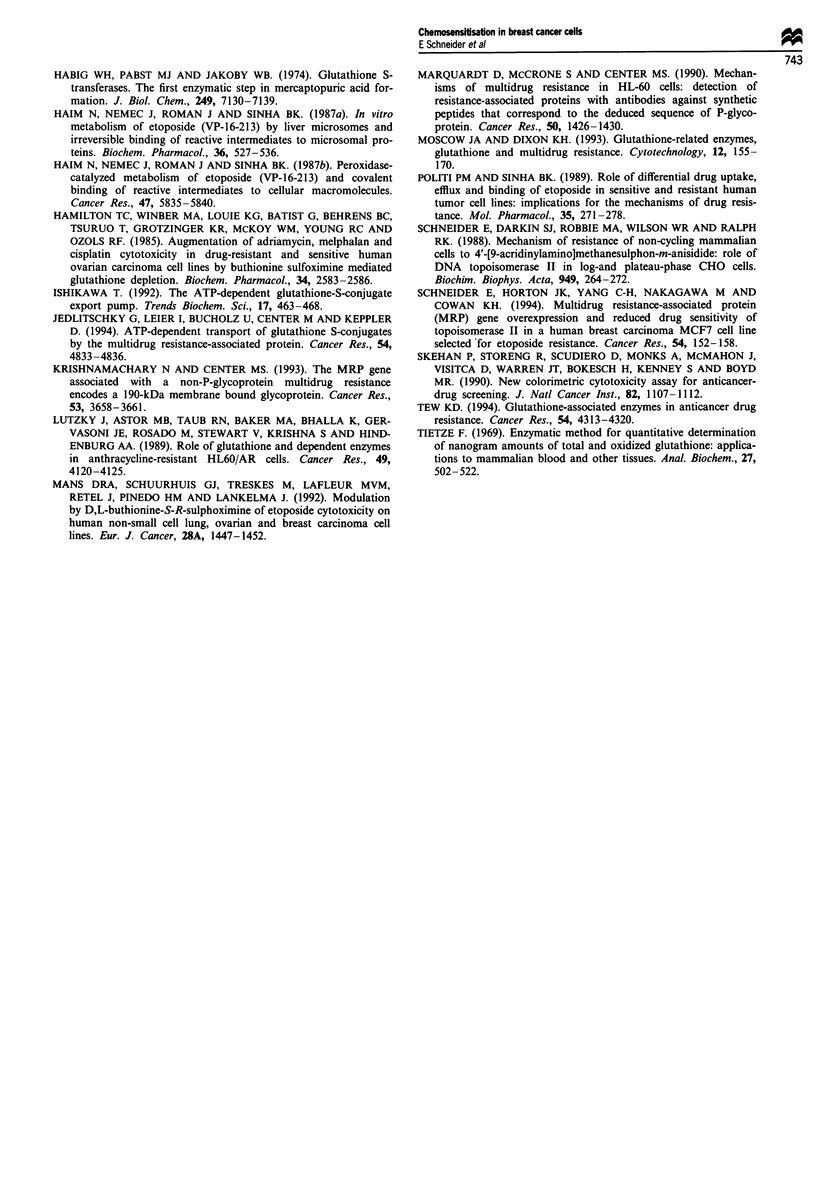# Buthionine sulphoximine-mediated sensitisation of etoposide-resistant human breast cancer MCF7 cells overexpressing the multidrug resistance-associated protein involves increased drug accumulation.

**DOI:** 10.1038/bjc.1995.144

**Published:** 1995-04

**Authors:** E. Schneider, H. Yamazaki, B. K. Sinha, K. H. Cowan

**Affiliations:** Medicine Branch, National Cancer Institute, Bethesda, MD 20892, USA.

## Abstract

Preincubation of etoposide-resistant human MCF7 breast cancer cells (MCF7/VP) with buthionine sulphoximine (BSO) resulted in their sensitisation to etoposide and vincristine. Chemosensitisation was accompanied by elevated intracellular drug levels. In contrast, simultaneous exposure to BSO did not result in increased drug accumulation. Similar, but quantitatively smaller, effects were also observed when sensitive wild-type MCF7/WT cells were treated with BSO. In agreement with its effect on drug accumulation, BSO pretreatment also increased VP-16-stimulated cleavable complex formation between DNA topoisomerase II and cellular DNA. BSO treatment also led to a significant increase in acid-precipitable VP-16 levels in MCF7/VP, but not MCF7/WT cells. In contrast, no clear effects of BSO on drug efflux were observed and drug retention was only minimally increased after BSO treatment of both MCF7/WT and MCF7/VP cells and no difference between the two cell lines was detected. Thus, chemosensitisation by BSO appeared to be mediated through increased intracellular drug concentrations and/or protein binding.


British Journal of Cancer (1995) 71, 738-743
© 1995 Stockton Press All rights reserved 0007-0920/95 $12.00

### Buthionine sulphoximine-mediated sensitisation of etoposide-resistant human breast cancer MCF7 cells overexpressing the multidrug resistance-associated protein involves increased drug accumulation

E Schneider[1], H Yamazaki[2,*], BK Sinha[2] and KH Cowan[1]

[1]*Medicine Branch and* [2]*Clinical Pharmacology Branch, National Cancer Institute, Bethesda MD 20892, USA.*

**Summary** Preincubation of etoposide-resistant human MCF7 breast cancer cells (MCF7/VP) with buthionine sulphoximine (BSO) resulted in their sensitisation to etoposide and vincristine. Chemosensitisation was accompanied by elevated intracellular drug levels. In contrast, simultaneous exposure to BSO did not result in increased drug accumulation. Similar, but quantitatively smaller, effects were also observed when sensitive wild-type MCF7/WT cells were treated with BSO. In agreement with its effect on drug accumulation, BSO pretreatment also increased VP-16-stimulated cleavable complex formation between DNA topoisomerase II and cellular DNA. BSO treatment also led to a significant increase in acid-precipitable VP-16 levels in MCF7/VP, but not MCF7/WT cells. In contrast, no clear effects of BSO on drug efflux were observed and drug retention was only minimally increased after BSO treatment of both MCF7/WT and MCF7/VP cells and no difference between the two cell lines was detected. Thus, chemosensitisation by BSO appeared to be mediated through increased intracellular drug concentrations and/or protein binding.

**Keywords:** MRP; chemosensitisation; drug accumulation; multidrug resistance

Increased intracellular glutathione (GSH) levels as well as increased activity of GSH-dependent enzymes have been associated with multidrug resistance in some studies, whereas others have failed to find evidence for a role of GSH or its dependent enzymes in multidrug resistance (reviewed in Moscow and Dixon, 1993). However, several studies have shown that treatment of cells with buthionine sulphoximine (BSO), an inhibitor of γ-glutamylcysteine synthetase, results in the depletion of intracellular glutathione and increased sensitivity of cells to several drugs, including melphalan, anthracyclines and etoposide (Green *et al.*, 1984; Hamilton *et al.*, 1985; Dusre *et al.*, 1989; Lutzky *et al.*, 1989; Mans *et al.*, 1992). Studies with doxorubicin-resistant HL60 cells (HL60/AR) have demonstrated that BSO treatment enhances the sensitivity of the resistant cells to the anthracycline daunorubicin and that this is accompanied by an increase in intracellular drug accumulation and retention (Lutzky *et al.*, 1989; Gollapudi and Gupta, 1992). Similar results have also been reported with unselected drug-sensitive human lung, ovarian and breast carcinoma cells (Mans *et al.*, 1992). In contrast, BSO treatment does not enhance doxorubicin sensitivity in multidrug-resistant small-cell lung cancer H69AR cells, despite GSH depletion (Cole *et al.*, 1990). Thus, although pharmacological depletion of GSH results in sensitisation of some cells to cytotoxic drugs, it is unclear whether this is a direct consequence of GSH depletion or whether the two effects occur independently.

A doxorubicin-selected, multidrug-resistant MCF7 human breast carcinoma cell line (MCF7/ADR) with increased GSH *S*-transferase levels that exhibits significant cross-resistance to etoposide has been described (Batist *et al.*, 1986), suggesting that GSH-mediated mechanisms might contribute to etoposide resistance. However, these cells also highly overexpress *MDR*1, which can also result in cross-resistance to etoposide. We have recently described a VP-16-selected, multidrug-resistant MCF7 subline (MCF7/VP) that does not express *MDR*1 mRNA, but instead contains 15-fold elevated levels of mRNA for the multidrug resistance-associated protein MRP (Schneider *et al.*, 1994). These cells are 30 to 60-fold resistant to VP-16, 9-fold resistant to doxorubicin and 5- to 15-fold resistant to vincristine, and resistance is, at least in part, due to a reduction in intracellular drug concentrations. In the present study we examined the effects of BSO treatment on the drug sensitivity of MCF7/VP cells. These studies demonstrated that BSO pretreatment resulted in greater sensitivity of MCF7/VP cells to VP-16 and vincristine and that chemosensitisation is associated with an increase in intracellular drug accumulation.

### Materials and methods

### *Materials*

All materials were as previously described (Schneider *et al.*, 1994) unless otherwise stated. Buthionine sulphoximine was obtained from Sigma (St Louis, MO, USA) and a 50 mM stock solution was prepared in water. [$^3$H]VP-16 was obtained from Moravek (Brea, CA, USA) and [$^3$H]vincristine was obtained from NEN (Wilmington, DE, USA).

### *Cell lines*

The selection of VP-16-resistant MCF7 (MCF7/VP) human breast cancer cells and culture conditions of wild-type (MCF7/WT) and VP-16 resistant MCF7 (MCF7/VP) cells were previously described (Schneider *et al.*, 1994).

### *Growth inhibition assays*

Cell growth assays were generally performed over a period of 7 days, using the sulphorhodamine B assay (Skehan *et al.*, 1990) and $IC_{50}$ values were determined as previously described (Schneider *et al.*, 1994). 2.5 μM BSO was added from stock solutions as indicated at the same time as the cytotoxic drug. In some experiments, 50 μM BSO was added 24 h before addition of VP-16 or vincristine and cells were incubated for an additional 24 h together with the cytotoxic drug. Thereafter, the drug-containing medium was removed and replaced with fresh drug- and BSO-free medium. Drug effects were then determined after a further 6 days' incubation. Under either of these conditions, survival of the cells in the presence of BSO alone was greater than 80%.

Correspondence: E Schneider
*Present address: Jikei University, 3-25-8 Nishi-Shinbashi, Minato-ku, Tokyo, Japan
Received 24 May 1994; revised 3 November 1994; accepted 7 December 1994

### Drug uptake, efflux and protein binding

Drug uptake and efflux studies were done essentially as described previously (Schneider et al., 1994). To study the effect of BSO on drug uptake, MCF7/WT and MCF7/VP cells were incubated for 2 h with 100 μM [$^3$H]VP-16 (1 μCi ml$^{-1}$) or 31 nM [$^3$H]vincristine (0.25 μCi ml$^{-1}$) in the absence or presence of BSO in serum-free culture medium, after which time the cells were analysed for retained radioactivity as described. To study the effect of glutathione depletion on drug accumulation and drug efflux, the cells were preincubated with 2.5 μM or 50 μM BSO for 5 days or 24 h, respectively, before adding the radioactive drug. After incubation for 2 h with 100 μM [$^3$H]VP-16 (1 μCi ml$^{-1}$) or 31 nM [$^3$H]vincristine (0.25 μCi ml$^{-1}$) in serum-free medium, the cells were washed with ice-cold phosphate-buffered saline (PBS), further incubated with prewarmed serum-and drug-free medium for 0–60 min, after which time the cells were again washed in ice-cold PBS and either directly lysed and assayed for remaining radioactivity or acid precipitated with 10% trichloroacetic acid. The precipitates were solubilised with 1 M sodium hydroxide and remaining radioactivity determined by scintillation counting.

### Potassium/SDS precipitation assay

MCF7/WT and MCF7/VP cells were incubated with 1 μCi ml$^{-1}$ [$^3$H]thymidine for 3 days. Approximately $10^5$ radiolabelled cells were then placed into each well of a 24-well culture plate and incubated for 2–3 h to allow them to adhere. The cells were then further incubated for 1 h in the absence or presence of 50 μM VP-16, either with or without 2.5 μM BSO or 50 μM BSO to determine the effect of these modulators on cleavable complex formation. Some cells were also preincubated for 5 days with 2.5 μM BSO or for 24 h with 50 μM BSO before determining cleavable complex formation in the presence of the same amount of BSO. Cells were lysed and protein–DNA complexes formed measured as previously described (Schneider et al., 1988).

### Measurements of glutathione and glutathione transferase levels

Glutathione levels were assayed by the kinetic assay of Tietze (1969). Glutathione S-transferase activity was assayed by monitoring the conjugation of glutathione with 1-chloro-2,4-dinitrobenzene (Habig et al., 1974).

### Protein determination

Protein concentrations were determined by the method of Bradford, (1976).

### Results

### Effect of BSO on drug sensitivity and glutathione levels

Intracellular GSH levels have been suggested to affect the sensitivity of cells to cytotoxic drugs. Therefore, we examined the effect of BSO on VP-16- and vincristine-mediated growth inhibition in MCF7/WT and MCF7/VP cells. As shown in Table I, co-incubation with 2.5 μM BSO for 7 days decreased the $IC_{50}$ of VP-16 in MCF7/VP cells 2.2-fold, whereas the sensitivity of MCF7/WT cells remained essentially unchanged. The results were similar when vincristine was used as the cytotoxic drug instead of VP-16.

Since BSO is known to reduce the intracellular levels of GSH, we investigated whether BSO-mediated sensitisation of MCF7/VP cells was associated with GSH depletion. The data presented in Table II show that GSH levels in resistant MCF7/VP cells were approximately 25% lower than in sensitive MCF7/WT cells and that they were further reduced by 50–60% in MCF7/VP cells after continuous treatment with 2.5 μM BSO for 5 days. This result suggested that the sensitisation of MCF7/VP cells by BSO may be mediated through the depletion of GSH. To further study this possibility, cells were preincubated with 50 μM BSO for 24 h which resulted in an 80–90% reduction in GSH levels in both cell lines (Table II). When cells were preincubated for 24 h in the presence of 50 μM BSO, followed by 24 h co-incubation with 50 μM BSO and VP-16, MCF7/VP cells

**Table II** Glutathione, glutathione transferase and the effect of buthionine sulphoximine in MCF7/WT and MCF7/VP cells

| Cell line | BSO (μM) | GSH (nmol mg$^{-1}$) | GST (nmol mg$^{-1}$ min$^{-1}$) |
|---|---|---|---|
| MCF7/WT | 0 | 113.5 ± 16.4 | 22.9 (22.3,23.5) |
| | 2.5[a] | 54.4 (52.1,56.6) | 25.0 (28.8,21.1) |
| | 50[b] | 20.6 (28,13.1) | ND |
| MCF7/VP | 0 | 85.1 ± 4.7 | 20.0 (17.7,22.3) |
| | 2.5[a] | 34.7 (36.2,33.2) | 25.7 (23.6,27.9) |
| | 50[b] | 7.7 (6.8,8.6) | ND |

MCF7/WT and MCF7/VP cells were incubated for 5 days with [a]2.5 μM BSO[a], or for 24 h with 50 μM BSO[b] before assaying for GSH levels and GST activity as described in Materials and methods. Standard errors are given where more than two experiments were performed; otherwise high and low values are given. ND, not determined.

**Table I** Modulation of VP-16 and vincristine cytotoxicity by BSO in MCF7/WT and MCF7/VP cells

| Treatment | MCF7/WT $IC_{50}$ | MCF7/WT ± s.e. (nM) | MCF7/WT Fold sensitisation | MCF7/VP $IC_{50}$ | MCF7/VP ± s.e. (nM) | MCF7/VP Fold sensitisation | Fold resistance | Fold reduction in resistance |
|---|---|---|---|---|---|---|---|---|
| VP-16[a] | 79 | ±16 | 1 | 4830 | ±1190 | 1 | 61 | |
| VP-16[a] + 2.5 μM BSO | 62 | ±17 | 1.3 | 2220 | ±590 | 2.2 | 36 | 1.7 |
| VP-16[b] | 210 | ±64 | 1 | 22375 | ±3060 | 1 | 106 | |
| VP-16[b] + 50 μM BSO | 81 | ±20 | 2.6 | 4355 | ±430 | 5.1 | 54 | 2.0 |
| Vincristine[a] | 0.23 | ±0.06 | 1 | 3.7 | ±0.9 | 1 | 16 | |
| Vincristine[a] + 2.5 μM BSO | 0.13 | ±0.02 | 1.8 | 0.9 | ±0.25 | 4.1 | 6.6 | 2.4 |
| Vincristine[b] | 0.5 | ±0.11 | 1 | 24.7 | ±5.3 | 1 | 49 | |
| Vincristine[b] + 50 μM BSO | 0.15 | ±0.01 | 3.4 | 0.5 | ±0.18 | 49 | 3.4 | 14 |

Cells were incubated with VP-16 or vincristine in the presence or absence of BSO as described in Materials and methods. [a]2.5 μM BSO or water was added at the time of cytotoxic drug addition and cells were incubated in the presence of both agents for 7 days. [b]Cells were preincubated with or without 50 μM BSO alone for 24 h, followed by co-incubation with VP-16 or vincristine for another 24 h. After removal of BSO and cytotoxic drug, incubation was continued for 6 days in drug- and BSO-free medium. $IC_{50}$ values were then determined graphically from growth inhibition curves. Values given are means from at least three experiments ± standard errors.

became even more sensitised to VP-16 than with continuous co-treatment (Table I). Interestingly, while the sensitivity of MCF7/VP cells to VP-16 increased 5-fold, the sensitivity of MCF7/WT cells also increased 2-fold under these conditions. Furthermore, despite the sensitisation of MCF7/VP cells by BSO, the resistant cell line remained significantly less sensitive to VP-16 than parental MCF7/WT cells incubated in the absence of BSO. In contrast, MCF7/VP cells that were incubated with BSO became as sensitive to vincristine ($IC_{50}$ = 0.5 nM) as parental MCF7/WT cells incubated without BSO ($IC_{50}$ = 0.5 nM). Thus, when compared with parental MCF7/WT cells in the absence of BSO, BSO was able to completely reverse vincristine, but not VP-16, resistance of MCF7/VP cells. No significant differences in total GST activities between the two cell lines were observed when incubated either in the absence or in the presence of 2.5 μM BSO for 5 days (Table II).

**Figure 1** Effect of BSO on VP-16 (**a**) and vincristine (**b**) uptake into MCF7/WT and MCF7/VP cells. Exponentially growing MCF7/WT and MCF7/VP cells were incubated for 2 h with 100 μM [$^3$H]VP-16 or 31 nM [$^3$H]vincristine at 37°C in the presence or absence of 2.5 μM or 50 μM BSO, which was added at the same time as VP-16 or vincristine (acute treatment); to test the effect of GSH depletion, cells were preincubated for 5 days with 2.5 μM BSO or for 24 h with 50 μM BSO, followed by 2 h of co-incubation with VP-16 or vincristine and BSO. The cells were then washed and the remaining radioactivity determined by liquid scintillation counting. The results shown are the means ± s.e. of at least three experiments. *P*-values were determined by Student's *t*-test.

*Effect of BSO on drug uptake*

We had previously shown that VP-16 and vincristine accumulation in drug-resistant MCF7/VP cells is 2- to 3-fold lower than in sensitive parental MCF7/WT cells, indicating that a defect in drug accumulation is at least partially responsible for the observed drug resistance (Schneider *et al.*, 1994). To examine the possibility that the increased growth-inhibitory effect of VP-16 and vincristine in the presence of BSO is mediated through increased intracellular drug concentrations, the accumulation of VP-16 and vincristine was measured in both cell lines in the absence or presence of 2.5 μM or 50 μM BSO. As shown in Figure 1a and 1b, incubation of MCF7/WT and MCF7/VP cells with VP-16 or vincristine for 2 h in the presence of 2.5 μM or 50 μM BSO (acute treatment) had little effect on intracellular drug levels, suggesting that BSO by itself did not directly affect drug accumulation. However, since growth inhibition was determined under conditions of reduced GSH levels, it was possible that drug accumulation was only altered in GSH-depleted cells. Indeed, after depletion of GSH by preincubation of cells for 5 days with 2.5 μM BSO or for 24 h with 50 μM BSO, intracellular drug levels were 40–60% higher in MCF7/VP cells, whereas no significant increase in drug accumulation was observed in MCF7/WT cells pretreated with BSO (Table III). Thus, it appeared that the increased sensitivity of MCF7/VP cells to cytotoxic drugs following prolonged exposure to BSO was associated with decreased intracellular GSH and increased intracellular drug concentration.

*Protein–DNA complex formation*

Cytotoxicity of VP-16 is mediated through the dose-dependent stimulation of cleavable complex formation between DNA topoisomerase II and the cellular DNA. We therefore examined whether the increase in intracellular VP-16 concentration after preincubation with BSO would also result in an increased formation of drug-induced protein–DNA complexes. As shown in Figure 2, increased cleavable complex formation was only observed when the cells had been preincubated with BSO, concomitant with the elevated intracellular drug levels. In contrast, there was no direct effect of BSO on VP-16-stimulated cleavable complex formation. This result was consistent with the hypothesis that the sensitisation of MCF7/VP cells by BSO pretreatment was associated with an increase in intracellular drug concentration and concomitant increase in drug-induced topoisomerase II-mediated DNA strand breaks. Furthermore, in light of the reduced susceptibility of topoisomerase II in the resistant cells to cleavable complex formation (Schneider *et al.*, 1994), this increase in cleavable complex formation is likely to be significant for the cells' drug sensitivity.

*Effect of BSO on drug efflux, retention and protein binding*

In order to determine whether the elevated drug levels found in MCF7/VP cells following BSO pretreatment were due to a reduction in drug efflux or due to increased drug retention, cells were preincubated with or without 2.5 μM BSO or 50 μM BSO alone for 5 days or 24 h, respectively, followed by incubation for 2 h with radioactive VP-16. After washing in ice-cold PBS, the cells were incubated for an additional 0–60 min in drug-free medium. At the indicated times total and acid-precipitable radioactivity remaining was then determined. As shown in Figure 3, there was no clear difference in drug efflux between untreated cells and cells that had been pretreated with BSO, although a small effect of 50 μM BSO pretreatment on VP-16 efflux from MCF7/VP cells cannot be

**Table III** The effect of two different BSO regimens on VP-16 and vincristine accumulation and sensitivity in MCF7/WT and MCF7/VP cells

| *Cell line* | *BSO* (μM) | *Relative GSH levels (%)* | *Relative drug levels VP-16* | *Relative drug levels Vincristine* | *Fold sensitisation VP-16* | *Fold sensitisation Vincristine* |
|---|---|---|---|---|---|---|
| MCF7/WT | 0 | 100 | 100 | 100 | 1 | 1 |
| | 2.5[a] | 48 | 100 | 114 | 1.3 | 1.8 |
| | 50[b] | 18 | 106 | 105 | 2.6 | 3.4 |
| MCF7/VP | 0 | 100 | 100 | 100 | 1 | 1 |
| | 2.5[a] | 41 | 135 | 132 | 2.2 | 4.1 |
| | 50[b] | 9 | 163 | 148 | 5.1 | 49 |

[a]2.5 μM BSO was added at the time of cytotoxic drug addition and cells were incubated in the presence of both agents for 7 days (growth inhibition assay) or were preincubated for 5 days before measuring GSH levels and drug accumulation. [b]Cells were preincubated with 50 μM BSO alone for 24 h before measuring GSH levels or drug accumulation, or co-incubated with VP-16 or vincristine for another 24 h followed by 6 days without BSO and cytotoxic drug (growth inhibition assay).

**Figure 2** Effect of BSO on VP-16-induced cleavable complex formation in MCF7/WT and MCF7/VP cells. The formation of DNA topoisomerase II–DNA cleavable complexes in MCF7/WT and MCF7/VP cells induced by a 1 h incubation with 50 μM VP-16 in the presence or absence of 2.5 μM or 50 μM BSO was examined by K/SDS precipitation assay. For acute treatment BSO was added at the same time as VP-16; to test the effect of GSH depletion, cells were preincubated for 5 days with 2.5 μM BSO or for 24 h with 50 μM BSO, followed by 1 h co-incubation with VP-16 and BSO. The results shown are the means ± s.e. of three experiments. *P*-values were determined by Student's *t*-test.

**Figure 3** Effect of BSO on VP-16 efflux from MCF7/WT and MCF7/VP cells. Cells were pretreated with 2.5 μM BSO for 5 days or 50 μM BSO for 24 h, followed by 2 h incubation with 100 μM [$^3$H]VP-16. After washing in ice-cold PBS, the cells were further incubated in drug-free medium. At the indicated times, the cells were lysed and remaining radioactivity determined by liquid scintillation counting. MCF7/WT: (■) no BSO; (◇) 2.5 μM BSO; (○) 50 μM BSO. MCF7/VP: (▲) no BSO; (□) 2.5 μM BSO; (▽) 50 μM BSO.

excluded. Furthermore, following BSO treatment total retained radioactivity after 1 h in both cell lines was not significantly increased. In contrast, a statistically significant increase (35%, $P = 0.04$) in acid-precipitable VP-16 was detected in MCF7/VP cells, whereas the amount of tightly bound drug remained unchanged in MCF7/WT cells after BSO treatment (Figure 4).

### Discussion

Chemosensitisation of cells by BSO is generally associated with glutathione depletion and is thought to be related to changes in the metabolism of cytotoxic drugs. However, some studies have also examined the possible effect of BSO on drug accumulation and/or intracellular drug distribution. For example, Lutzky *et al.* (1989) investigated the effect of BSO on glutathione and drug levels as well as on intracellular drug distribution in a doxorubicin-resistant HL60 cell line, HL60/AR, which has been shown to overexpress p190, the product of the MRP gene (Marquardt *et al.*, 1990; Krishnamachary and Center, 1993). Following pretreatment with BSO, drug sensitivity as well as intracellular accumulation and retention of daunorubicin were increased in the resistant but not the sensitive parental cells, although GSH levels were similarly reduced in both cell lines. Mans *et al.* (1992) have shown that in MCF7, A2780 and SW-1573 cells BSO treatment reduces GSH concentrations in a time-dependent manner, accompanied by increased intracellular VP-16 levels and cytotoxicity. These authors also suggested that the effect of BSO on VP-16 accumulation is not due to a direct inhibition of drug transport by BSO. Similarly, in the current study we found that BSO had no direct effect on drug accumulation or

**Figure 4** Effect of BSO on VP-16 retention and protein binding. Cells were pretreated with or without 50 μM BSO for 24 h, followed by 2 h in the presence of 50 μM BSO and 100 μM [$^3$H]VP-16. After a further 1 h in the absence of VP-16, the cells were analysed for remaining radioactivity (drug retention) and acid-precipitable radioactivity (protein binding).

efflux. Rather, pretreatment of the resistant cells with BSO was required in order for BSO to affect drug accumulation. Although the BSO-mediated increase in drug accumulation was accompanied by increased drug-mediated growth inhibition and GSH depletion in MCF7/VP cells, only minimally higher drug levels and drug sensitivity were observed in MCF7/WT cells, despite similar GSH depletion (Table III). These results suggest that GSH depletion may be necessary but not sufficient for increased drug sensitivity and accumulation. However, MCF7/VP cells contained lower basal levels of GSH than the parental MCF7/WT cells, suggesting that, in addition to decreased drug accumulation, resistance in MCF7/VP cells may also be associated with lower GSH levels. Similarly, decreased GSH levels were also noted in drug-resistant HL60/AR and H69AR cells, both of which had GSH levels 2- to 6-fold lower than their drug-sensitive parental cell lines (Lutzky *et al.*, 1989; Cole *et al.*, 1990). Thus, it remains unclear what, if any, role pharmacological GSH depletion may have in altering drug sensitivity and accumulation in MCF7/VP cells.

Studies by Haim. (1987*a*, *b*) have shown that metabolites of VP-16 can covalently bind to proteins and DNA, and it was suggested that the bound metabolites are reactive and potentially damaging and capable of causing cellular injury and cell death. Thus, it is possible that the relative increase in acid-precipitable VP-16 in MCF7/VP cells after BSO treatment was, at least in part, responsible for the increased cytotoxicity. The non-effluxable pool of VP-16 in sensitive MCF7/WT cells was 3-fold higher than that in resistant MCF7/VP cells ($135 \pm 10$ *vs* $47 \pm 2$ pmol of bound VP-16 per mg of protein for sensitive and resistant cells respectively). A similar difference was also observed in HL60 and HL60/AR cells (Politi and Sinha, 1989). Upon incubation with BSO, there was a 35% increase in the non-effluxable pool in MCF7/VP cells, whereas the amount of bound drug remained unchanged in MCF7/WT cells. Consequently, the ratio of bound VP-16 between sensitive MCF7/WT and resistant MCF7/VP cells decreased from 3- to 2-fold upon BSO treatment ($144 \pm 23$ *vs* $63 \pm 6$ pmol of bound VP-16 per mg of protein for sensitive and resistant cells respectively). Thus, it is conceivable that the increased sensitisation of MCF7/VP cells by BSO resulted from an increase in the pool of non-effluxable VP-16.

Alternative mechanisms for BSO-mediated increases in drug sensitivity of cells have been proposed. For example, Mans *et al.* (1992) have suggested that BSO may cause membrane alterations which may affect cellular drug efflux. However, when Gollapudi and Gupta (1992) measured the effect of BSO on plasma membrane potential in HL60 and HL60/AR cells they were unable to detect any alterations, although the concentration of BSO and the time of exposure used in these experiments is unclear. Thus, it is not clear whether BSO can directly affect the plasma membrane. In contrast, Lutzky *et al.* (1989) suggested in their study that BSO might have affected intracellular distribution of GSH/GST, which in turn may have led to increased drug accumulation and retention. Whether these two effects were related or independent remained unanswered. We have at present no evidence that either of these potential mechanisms contributed to the BSO-mediated chemosensitisation of MCF7/VP cells.

It was recently suggested that the multidrug resistance-associated protein (MRP), which is overexpressed in the MCF7/VP cells, functions as an efflux pump for glutathione-conjugated compounds (GS-X) (Ishikawa, 1992; Jedlitschky *et al.*, 1994). Thus, one might expect that depletion of GSH would lead to a reduction in the amount of conjugated substrate for GS-X and consequently result in higher intracellular drug concentrations. However, it is unclear if such a mechanism is responsible for the observed sensitisation of MCF7/VP cells by BSO, since neither VP-16 nor vincristine is a substrate for GSH conjugation (Tew, 1994).

In conclusion, we have shown that BSO is able to sensitise resistant MCF7/VP cells and that this effect is mediated through an increase in intracellular drug concentration. Our results suggest that GSH depletion may be necessary but not sufficient for increased drug sensitivity and accumulation. In addition, increased drug binding in the resistant cells may also contribute to their sensitisation. However, additional experiments are required to define further the mechanism(s) involved in BSO-mediated chemosensitisation of MCF7/VP cells.

**Abbreviations:** P-gp, P-glycoprotein; MRP, multidrug resistance-associated protein; WT, wild-type; MDR, multidrug resistance; s.e., standard error; PBS, phosphate-buffered saline; $IC_{50}$, drug concentration that inhibited cell growth by 50% under the assay conditions used; BSO, buthionine sulphoximine.

### References

BATIST G, TULPULE A, SINHA BK, KATKI AG, MYERS CE AND COWAN KH. (1986). Overexpression of a novel anionic glutathione transferase in multidrug resistant human breast cancer cells. *J. Biol. Chem.*, **261**, 15544–15549.

BRADFORD MM. (1976). A rapid and sensitive method for the quantitation of microgram quantities of protein utilizing the principal of protein-dye binding. *Anal. Biochem.*, **72**, 248–254.

COLE SPC, DOWNES HF, MIRSKI SEL AND CLEMENTS DJ. (1990). Alterations in glutathione and glutathione related enzymes in a multidrug-resistant small cell lung cancer cell line. *Mol. Pharmacol.*, **37**, 192–197.

DUSRE L, MIMNAUGH EG, MYERS CE AND SINHA BK. (1989). Potentiation of doxorubicin cytotoxicity by buthionine sulfoximine in multidrug-resistant human breast tumor cells. *Cancer Res.*, **49**, 511–515.

GOLLAPUDI S AND GUPTA S. (1992). Lack of reversal of daunorubicin resistance in HL60/AR cells by cyclosporin A. *Anticancer Res.*, **12**, 2127–2132.

GREEN JA, VISTICA DT, YOUNG RC, HAMILTON TC, ROGAN AM AND OZOLS RF. (1984). Potentiation of melphalan cytotoxicity in human ovarian cancer cell lines by glutathione depletion. *Cancer Res.*, **44**, 5427–5431.

HABIG WH, PABST MJ AND JAKOBY WB. (1974). Glutathione S-transferases. The first enzymatic step in mercaptopuric acid formation. *J. Biol. Chem.*, **249**, 7130–7139.

HAIM N, NEMEC J, ROMAN J AND SINHA BK. (1987*a*). *In vitro* metabolism of etoposide (VP-16-213) by liver microsomes and irreversible binding of reactive intermediates to microsomal proteins. *Biochem. Pharmacol.*, **36**, 527–536.

HAIM N, NEMEC J, ROMAN J AND SINHA BK. (1987*b*). Peroxidase-catalyzed metabolism of etoposide (VP-16-213) and covalent binding of reactive intermediates to cellular macromolecules. *Cancer Res.*, **47**, 5835–5840.

HAMILTON TC, WINBER MA, LOUIE KG, BATIST G, BEHRENS BC, TSURUO T, GROTZINGER KR, MCKOY WM, YOUNG RC AND OZOLS RF. (1985). Augmentation of adriamycin, melphalan and cisplatin cytotoxicity in drug-resistant and sensitive human ovarian carcinoma cell lines by buthionine sulfoximine mediated glutathione depletion. *Biochem. Pharmacol.*, **34**, 2583–2586.

ISHIKAWA T. (1992). The ATP-dependent glutathione-S-conjugate export pump. *Trends Biochem. Sci.*, **17**, 463–468.

JEDLITSCHKY G, LEIER I, BUCHOLZ U, CENTER M AND KEPPLER D. (1994). ATP-dependent transport of glutathione S-conjugates by the multidrug resistance-associated protein. *Cancer Res.*, **54**, 4833–4836.

KRISHNAMACHARY N AND CENTER MS. (1993). The MRP gene associated with a non-P-glycoprotein multidrug resistance encodes a 190-kDa membrane bound glycoprotein. *Cancer Res.*, **53**, 3658–3661.

LUTZKY J, ASTOR MB, TAUB RN, BAKER MA, BHALLA K, GERVASONI JE, ROSADO M, STEWART V, KRISHNA S AND HINDENBURG AA. (1989). Role of glutathione and dependent enzymes in anthracycline-resistant HL60/AR cells. *Cancer Res.*, **49**, 4120–4125.

MANS DRA, SCHUURHUIS GJ, TRESKES M, LAFLEUR MVM, RETEL J, PINEDO HM AND LANKELMA J. (1992). Modulation by D,L-buthionine-*S*-*R*-sulphoximine of etoposide cytotoxicity on human non-small cell lung, ovarian and breast carcinoma cell lines. *Eur. J. Cancer*, **28A**, 1447–1452.

MARQUARDT D, MCCRONE S AND CENTER MS. (1990). Mechanisms of multidrug resistance in HL-60 cells: detection of resistance-associated proteins with antibodies against synthetic peptides that correspond to the deduced sequence of P-glycoprotein. *Cancer Res.*, **50**, 1426–1430.

MOSCOW JA AND DIXON KH. (1993). Glutathione-related enzymes, glutathione and multidrug resistance. *Cytotechnology*, **12**, 155–170.

POLITI PM AND SINHA BK. (1989). Role of differential drug uptake, efflux and binding of etoposide in sensitive and resistant human tumor cell lines: implications for the mechanisms of drug resistance. *Mol. Pharmacol.*, **35**, 271–278.

SCHNEIDER E, DARKIN SJ, ROBBIE MA, WILSON WR AND RALPH RK. (1988). Mechanism of resistance of non-cycling mammalian cells to 4′-[9-acridinylamino]methanesulphon-*m*-anisidide: role of DNA topoisomerase II in log-and plateau-phase CHO cells. *Biochim. Biophys. Acta*, **949**, 264–272.

SCHNEIDER E, HORTON JK, YANG C-H, NAKAGAWA M AND COWAN KH. (1994). Multidrug resistance-associated protein (MRP) gene overexpression and reduced drug sensitivity of topoisomerase II in a human breast carcinoma MCF7 cell line selected for etoposide resistance. *Cancer Res.*, **54**, 152–158.

SKEHAN P, STORENG R, SCUDIERO D, MONKS A, MCMAHON J, VISITCA D, WARREN JT, BOKESCH H, KENNEY S AND BOYD MR. (1990). New colorimetric cytotoxicity assay for anticancer-drug screening. *J. Natl Cancer Inst.*, **82**, 1107–1112.

TEW KD. (1994). Glutathione-associated enzymes in anticancer drug resistance. *Cancer Res.*, **54**, 4313–4320.

TIETZE F. (1969). Enzymatic method for quantitative determination of nanogram amounts of total and oxidized glutathione: applications to mammalian blood and other tissues. *Anal. Biochem.*, **27**, 502–522.